# Supplementary material for: Genetic Analysis of Torque Teno Canis Virus Identified in Republic of Korea
Source: Vet Sci. 2022 Dec 13;9(12):693. doi: 10.3390/vetsci9120693 (PMC9782174; doi:10.3390/vetsci9120693)
Supplement: Supplementary file 1 [file vetsci-09-00693-s001.zip › vetsci-2040099-supplementary.pdf]

Figure S1. Phylogenetic tree generated using the partial sequences of TTCaV amplified by primer set 1 and the reference strains. The 13 TTCaV-positive samples identified in this study are marked with closed triangles

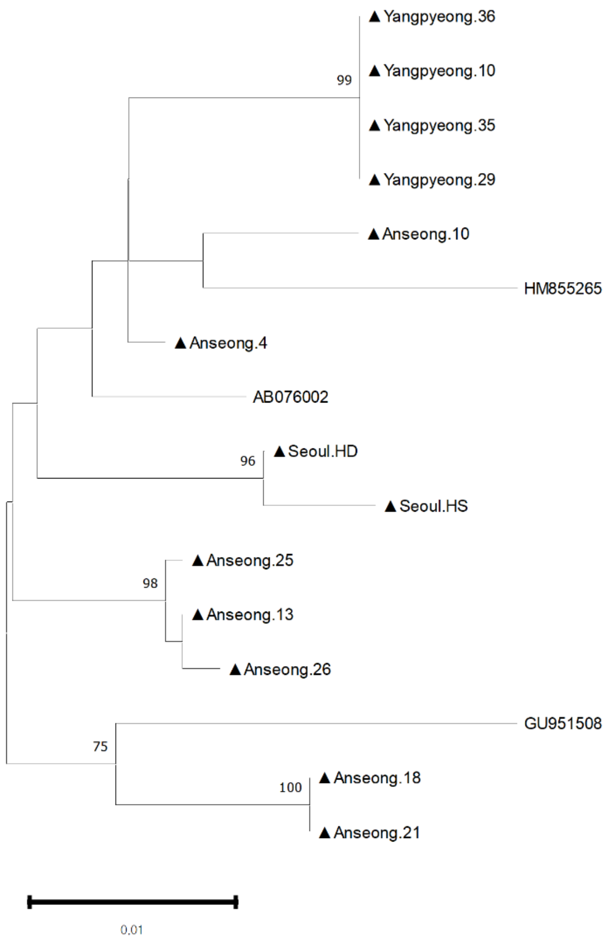

Table S1. Recombination region identified in MZ503912

Recombinant	Parental virus		Breakpoints[a]		Score for the seven detection methods in RDP4[b]						
Virus	Major	Minor	Begin	End	RDP	GENECONV	BootScan	MaxChi	Chimaera	SiScan	3Seq
MZ503912	KX827768	MZ503910	40	985	1.3×10^{-224}	1.1×10^{-226}	5.5×10^{-223}	1.2×10^{-60}	3.6×10^{-42}	4.8×10^{-83}	6.3×10^{-14}

[a] The breakpoints are based on the locations in the genome of MZ503912.

[b] The p value cut-off was 0.05. $p < 0.05$ indicated that the recombination events were significant.
